# Catapol reduced the cognitive and mood dysfunctions in post-stroke depression mice via promoting PI3K-mediated adult neurogenesis

**DOI:** 10.18632/aging.204979

**Published:** 2023-08-29

**Authors:** Bo Li, Xin Zhou, Lu Zhen, Weiwei Zhang, Jian Lu, Jie Zhou, Huoquan Tang, Huangsuo Wang

**Affiliations:** 1 Department of Neurosurgery, General Hospital of Taiyuan Iron and Steel Co, Taiyuan, China; 2 Medical Laboratory, Shanxi Province Pediatric Hospital, Taiyuan, China; 3 Department of Endocrinology, General Hospital of Taiyuan Iron and Steel Co, Taiyuan, China; 4 Department of Anesthesiology, Shanxi Bethune Hospital, Taiyuan, China

**Keywords:** post stroke depression, catapol, adult hippocampal neurogenesis, PI3K

## Abstract

Adult hippocampal neurogenesis provides a regenerative resource for neural tissue and enhances neural plasticity, which is beneficial for brain functional rehabilitation post stroke. Recently, an increasing number of metabolic drugs have been reported to attenuate behavioral symptoms in neurodegeneration or psychiatric disorders via promoting adult hippocampal neurogenesis. Bioeffects of catapol show its potential as an antidiabetic though it has been previously widely indicated to perform the neuroprotective functions. However, the systematic evidence to support the behavioral effects of catapol to PSD model and what is the role of adult neurogenesis in such effects remains unexplored. In current study, we created the PSD model by combining MCAO procedure and CORT feeding. The treatment of catapol strikingly reduced the depressive/anxiety behavior in PSD model. Moreover, treatment of catapol also improved the cognitive functions. Immunofluorescence indicates that catapol could promote adult hippocampal neurogenesis in PSD model, and TMZ treatment further confirmed the role of the hippocampal neurogenesis in catapol’s therapeutic effects to PSD. Cultural neurons also indicates that PI3K is the key signal in regulating catapol mediated neurogenesis. By administrating the PI3K specific inhibitor, we found that PI3K is the key to mediate the behavioral effects of catapol to PSD. In conclusion, catapol could perform as the effective drug to treat PSD via the PI3K mediated adult hippocampal neurogenesis.

## INTRODUCTION

Stroke particularly ischemic stroke usually results in the serious brain damage and the prolonged neural dysfunctions to affect the daily life of the patients [1]. Depression is the most frequent psychiatric symptom following ischemic stroke. Apart from suffering from panic, patients with post-stroke depression (PSD) usually have negative functional outcome upon survival, delayed rehabilitation and decreased quality of life [2]. Several lines of evidence indicate that administration of antidepressants such as selective seratonin reuptake inhibitors (SSRIs) could benefit the post stroke brain recovery [3]. Therefore, preventing the development of PSD is the critical therapeutic task to post stroke rehabilitation.

Adult hippocampal neurogenesis is a lifelong process initiated by adult neural stem cells in the hippocampal dentate gyrus (DG) region [4]. Adult neural stem cells undergoing self-renewal and neural lineage commitment would form into immature neurons and mature functional neurons to integrate into the hippocampal circuit. Adult hippocampal neurogenesis are widely considered as one of main brain functions not only support the intrinsic regeneration but also the structural plasticity to facilitate the brain to adapt the environmental stress. The new formed neurons was reported to act critically in regulating antidepressant behaviors [5]. Therefore, exploring the new compounds with the neurogenic promotion functions would help the development of new drugs in neural repairing during post-stroke. There are many natural compounds that extracted from traditional Chinese Medical herbs have been demonstrated can perform the roles in regulating hippocampal neural functions. Previous report indicated that catapol could reduce the neurological dysfunctions in stroke model [6]. However, whether catapol could exert the long-term improvement to post-stroke brain and play as the therapeutic agent to PSD remains unidentified. If the hippocampal neurogenesis is the prerequisite for the effects of catapol to improve the neurological functions after stroke is yet to known.

It was widely reported that metabolic regulators play the pivotal role in regulating adult hippocampal neurogenesis [7]. Metabolic factors particularly insulin pathway are widely reported to mediate the neural differentiation of the neural stem cells *in vivo* and *in vitro* [8, 9]. Key signaling proteins act in regulating insulin or insulin growth factor pathway also mediate stem cell behaviors including the proliferation, differentiation and survive of the neural stem/progenitor cells [10]. PI3K signaling pathway was also reported as the key molecular mechanism underlying the pro-neurogenic effects of the antidepressant [11]. It was also demonstrated that catapol could ameliorates hepatic insulin resistance in type 2 diabetes through acting on PI3K/Akt pathway [12]. Hence, in current study we further analysis the role of the PI3K in the pro-neurogenic effects as well as the behavioral protection of catapol in PSD mice.

## MATERIALS AND METHODS

### Animals

Animals were housed in groups of four or five and maintained in standard conditions (controlled room temperature and humidity, 12 h/12 h light/dark cycle, with lights on at 8:00 A.M., ad libitum access to dry food pellets and water). Anesthesia was achieved using nose cone-delivered isoflurane (maintained at 1.5% in 80% N2O and 20% O2). MCAO was induced by a silicon rubber-coated 7-0 monofilament (Doccol Corporation, Redlands, CA) in the internal carotid artery, after which the monofilament was advanced to occlude the MCA. The filament was withdrawn 45 min after occlusion, and reperfusion was confirmed by laser Doppler monitoring. The surgical wound was sutured, and mice could recover from anesthesia. Mice were anesthetized with halothane (1.5 to 2% in O2-enriched air by face mask) and kept warm on water pads.

With a 5 days habituation, mice were then fed with corticosterone (CORT, 70μg/ml in drinking water) for 2 weeks for inducing post-stroke depression, during which the survive of the mice were recorded for evaluating the drug effects. For drug treatment, catapol with two dosages (5mg/kg, 20mg/kg) as well as the clinical medication control fluoxetine (FLX, 10mg/kg) and piracetam (75mg/kg) were administrated to the subjects daily with IP injection. The dosage of FLX and piracetam were followed with previous reports [13, 14]. The treatment was performed simultaneous with the CORT administration. For pre-temozolomide (TMZ), TMZ (2.5mg/kg) was treated to the mouse by IP for 3 days before the MCAO performance. For post-TMZ treatment, the mice were received with TMZ (2.5mg/kg) for 3 days alongside with the catapol administration from the treatment of CORT. For pre-LY294002, LY294002 (5mg/kg) was treated to the mouse by IP for 3 days before the MCAO performance. For post-LY294002 treatment, the mice were received with LY294002 (5mg/kg) for 3 days alongside with the catapol administration from the treatment of CORT.

### Behavioral tests

Mice in all groups were conducted with behavioral test the day after the drug treatments. The mood behaviors were evaluated with forced swim test (FST), splash test, open field test (OFT) and sucrose preference test (SPT). While the cognitive performance of the mice was reflected in the objective recognition test (ORT).

### Forced swim test

Mice were put into a cylinder water tank (30 cm height × 20 cm diameters) with the water and allow the free swimming for 6min. Whole behavior test process was video recorded, and mobility time within last 4 min (define the first 2 min as habituation) including struggling and free swim time were recorded for analyzing.

### Splash test

In splash test, 10% of the sucrose water solution was splashed on the back of the mouse and behavior of the mice in 10min after the splash were video recorded. During the test, a far-red light was used for warming the back of the mouse, and the grooming time of the mouse was recorded for analyzing its antidepressant ability.

### Open field test

Mice were put into an open field with the size of 43cm × 43cm for 10min. Mice could freely move in the arena, which was artificially separated by 16 equal squares. Duration of the animal traveled in the central area was recorded to analyze the anxiety level. Open field would be cleaned with 75% ethanol before the test.

### Sucrose preference test

For SPT, mouse was single caged and fed with two bottles of water, one with normal tape water and another with 2% of sucrose solution dissolved in tap water. For first 3 days, the consumption of the sucrose water percentage was recorded as the preference index. Then the mouse was conducted with food and water deprivation for 18h as the stress. Afterward, two bottles of water were provided again and recorded the preference index in 1h.

### Objective recognition test

24h after OFT, the mouse was put into the open field again with the two identical same toys (3cm^3^ blue cube) and allowed the free exploring for 10min or until the total exploration time to the toys over 20 sec. The explore behavior was identified as the nose near the toy within 2cm. After the learning period, the mouse was put out and stay with an interval of 5h. Then put the mouse back into the open field with one original toy and another new toy (3cm diameter, 3cm height red cylinder). The mouse was allowed for free explore in the open field. During the 10 min test, the mouse was taken out when total exploration time over 20 sec. The recognition index was calculated as exploration time to new object/total exploration time.

### Immunofluorescence

Brain cyrosections and cell cultural slices on cover slips were prepared. Primary antibodies (Rabbit-anti-DCX, CST, 1:400) were incubated with sections at 4° C overnight. Secondary antibodies (Goat-anti-Rabbit-Alexa fluor 488, Thermo Fisher, 1:800) were incubated with sections for 2h at RT. DAPI (Sigma) was incubated with sections for 10min. Tissue IF images were obtained with confocal microscopy (Nikon, C2+) with Z-stack for 20μm and projection on Z-stack was performed with ImageJ.

### Neural progenitor cells culture and induction

Briefly, fetal Sprague–Dawley rats (embryonic days E14-15). The dissociated cells (1 × 105 cells/ml) were suspended in DMEM/F12 medium replenished with 2% B27 supplement, recombinant human bFGF (20 ng/ml), and EGF (20 ng/ml) with B27 applied. NSCs were mechanically dissociated as single cells and directly plated onto poly-L-lysine coated coverslips in the culture medium withdraw growth factors for 7 days. The cell was treated with hypoxia for 48h following with 24h dexamethasone (10μM) administration to mimic PSD in neurons. Catapol (20μM) and LY294002 (10μM) was dissolved in the cultural medium for 24h. Then the cultural neurons were fixed and perform the IF test.

### Statistic

For comparison across multiple groups, statistical comparisons were performed by one-way analysis of variance (ANOVA) followed by multiple comparison test via GraphPad Prism 9 (GraphPad software Inc, CA, USA). For SPT analysis, two-way ANOVA following multiple comparison test was performed. A p value of 0.05 is considered as statistically significant. All values are expressed as mean ± SEM.

## RESULTS

### Catapol attenuates post stroke induced depressive symptoms

**Figure 1:**
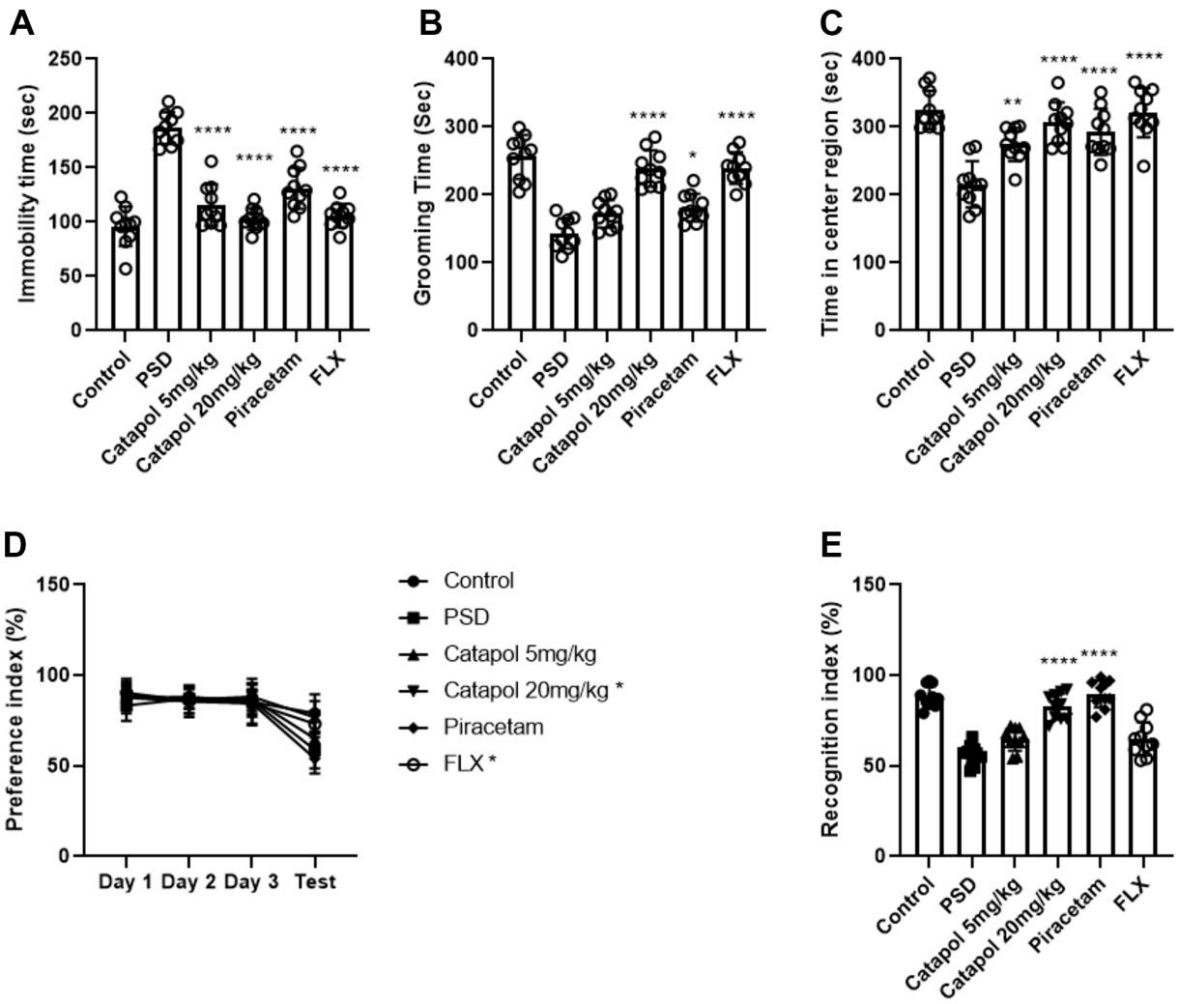
**Catapol attenuates post stroke induced depressive symptoms.** (**A**) Statistical analysis on the immobility time of the FST test. One-way ANOVA, Tukey’s post-hoc, compared with PSD: ****: p<0.001. (**B**) Statistical analysis on the grooming time in splash test. One-way ANOVA, Tukey’s post-hoc, compared with PSD: *: p<0.05, ****: p<0.001. (**C**) Statistical analysis on the time in center region of OFT. One-way ANOVA, Tukey’s post-hoc, compared with PSD: **: p<0.01, ****: p<0.001. (**D**) Statistical analysis on the preference index in SPT. Two-way ANOVA, Tukey’s post-hoc, compared with PSD: *: p<0.05. (**E**) Statistical analysis on the recognition index in ORT. One-way ANOVA, Tukey’s post-hoc, compared with PSD: ****: p<0.001.

We firstly investigated the behavioral effects of the catapol to PSD mouse. Catapol were treated to the mouse after MCAO with the dosage of 5mg/kg/d and 20mg/kg/d. Another two groups of mice were treated with frequent used brain recovery medications piracetam and SSRIs antidepressant fluoxetine (FLX) as 2 groups of positive control. Forced swim test (FST) was conducted for testing the learned helplessness behavior. Compared with PSD model, treatment of catapol with low (5mg/kg) and high (20mg/kg) dosages both reduced the immobility time in FST (Figure 1A, one-way ANOVA, p<0.001 vs. PSD group). Piracetam and FLX treatments gained the comparable effects as catapol (Figure 1A, one-way ANOVA, p<0.001 vs. PSD group). In splash test result, high dosage of catapol could significantly elevate the grooming behavioral as the piracetam and FLX did (Figure 1B, one-way ANOVA, catapol and FLX: p<0.001 vs. PSD group; Piracetam: p<0.05 vs. PSD), which suggested the dose dependent effects of catapol in alleviating the depression mood. Anxiety symptom was assessed with open field test (OFT). Compared with PSD group, treatment of both catapol with low and high dosages significantly increased the activity of mice in central region as the piracetam and FLX did (Figure 1C, one-way ANOVA, 5mg/kg/d vs. PSD: p<0.01; 20mg/kg/d, piracetam and FLX vs. PSD: p<0.001). The anhedonia was detected with the sucrose preference test (SPT). As the result showed, the preference index during habituation stage did not show any significant difference among groups. With the stress of 18h water deprivation, high dosage of catapol treatment and FLX improved the preference in compared with PSD group (Figure 1D, two-way ANOVA, catapol and FLX: p<0.05 vs. PSD). Moreover, in objective recognition test, high dosage of catapol treatment and piracetam improved the recognition index in compared with PSD group (Figure 1E, one-way ANOVA, catapol and piracetam: p<0.001 vs. PSD). Collectively, 20mg/kg/d catapol treatment could result in a stable improvement effect of depressive and cognitive behaviors in PSD model.

### Catapol treatment enhanced the hippocampal neurogenesis in PSD model

**Figure 2:**
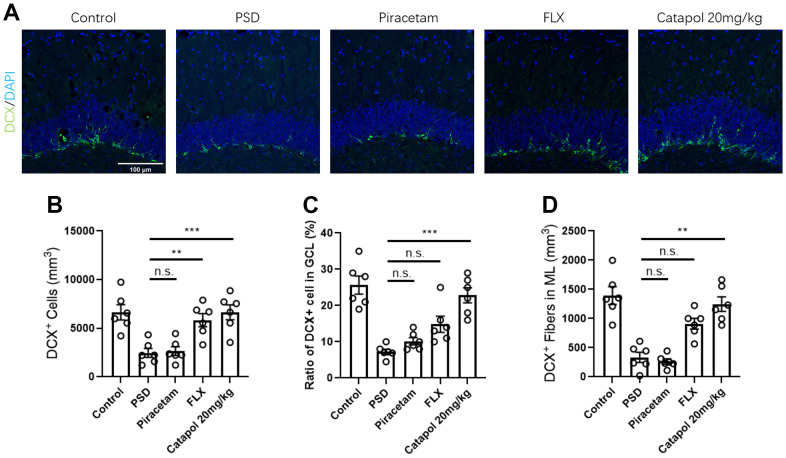
**Catapol perform the behavioral effects in PSD model in dependent with hippocampal neurogenesis.** (**A**) IF image to show the density of DCX^+^ immature neurons (Green) in DG region. DAPI (Blue) was labelled with blue color. (**B**) Statistical analysis of DCX^+^ immature neurons. One-way ANOVA, Tukey’s post-hoc. (**C**) Statistical analysis of the ratio of DCX^+^ immature neurons GCL region. One-way ANOVA, Tukey’s post-hoc. (**D**) Statistical analysis of the ratio of DCX^+^ immature neural fiber density in ML. One-way ANOVA, Tukey’s post-hoc.

We next tested the effects of catapol to adult neurogenesis via evaluating the tissue contribution of doublecortin (DCX) positive neuron at hippocampal, which was suggested as the hall mark of immature neurons [15]. Given that catapol treatment with 20mg/kg/d exerted the stable behavioral effects to PSD model, we compared effects of this dosage to other two commonly used drugs. As the result showed, PSD induced the significant decreased hippocampal neurogenesis that was reflected by the reduced density of DCX+ newborn neurons (Figure 2A, 2B; one-way ANOVA, Tukey’s post-hoc test, p<0.01 FLX vs. PSD; p<0.001 Control vs. PSD). Treatment of the catapol rescued the immature neuronal defects in PSD model (Figure 2A, 2B; one-way ANOVA, Tukey’s post-hoc test, p<0.01 FLX vs. PSD; p<0.001 Catapol vs. PSD). To further assess the migration of the newborn neurons, we analyzed the DCX^+^ fibers in the granular cell layer (GCL) region. Treatment of catapol remarkably increased the DCX^+^ fibers in the GCL in relative to PSD group (Figure 2A, 2C; one-way ANOVA, Tukey’s post-hoc test, p<0.001 Catapol vs. PSD). Such effect was absent from the hippocampus that administrated with piracetam and FLX (Figure 2A, 2C). We further detected the DCX^+^ fibers in the molecular layer (ML) for further assess the dendritic enrichment of the newborn neurons. Treatment of catapol remarkably increased the DCX^+^ fibers in the ML compared with PSD group (Figure 2A, 2D; one-way ANOVA, Tukey’s post-hoc test, p<0.01 Catapol vs. PSD). Such effect was not observed in neither piracetam nor FLX treatment group. Collectively, catapol could sufficiently improve the hippocampal neurogenesis in PSD animal model, which includes an enriched biological effects in compared with classic neuroprotective or antidepressant drugs.

### Catapol perform the behavioral effects in PSD model in dependent with hippocampal neurogenesis

**Figure 3:**
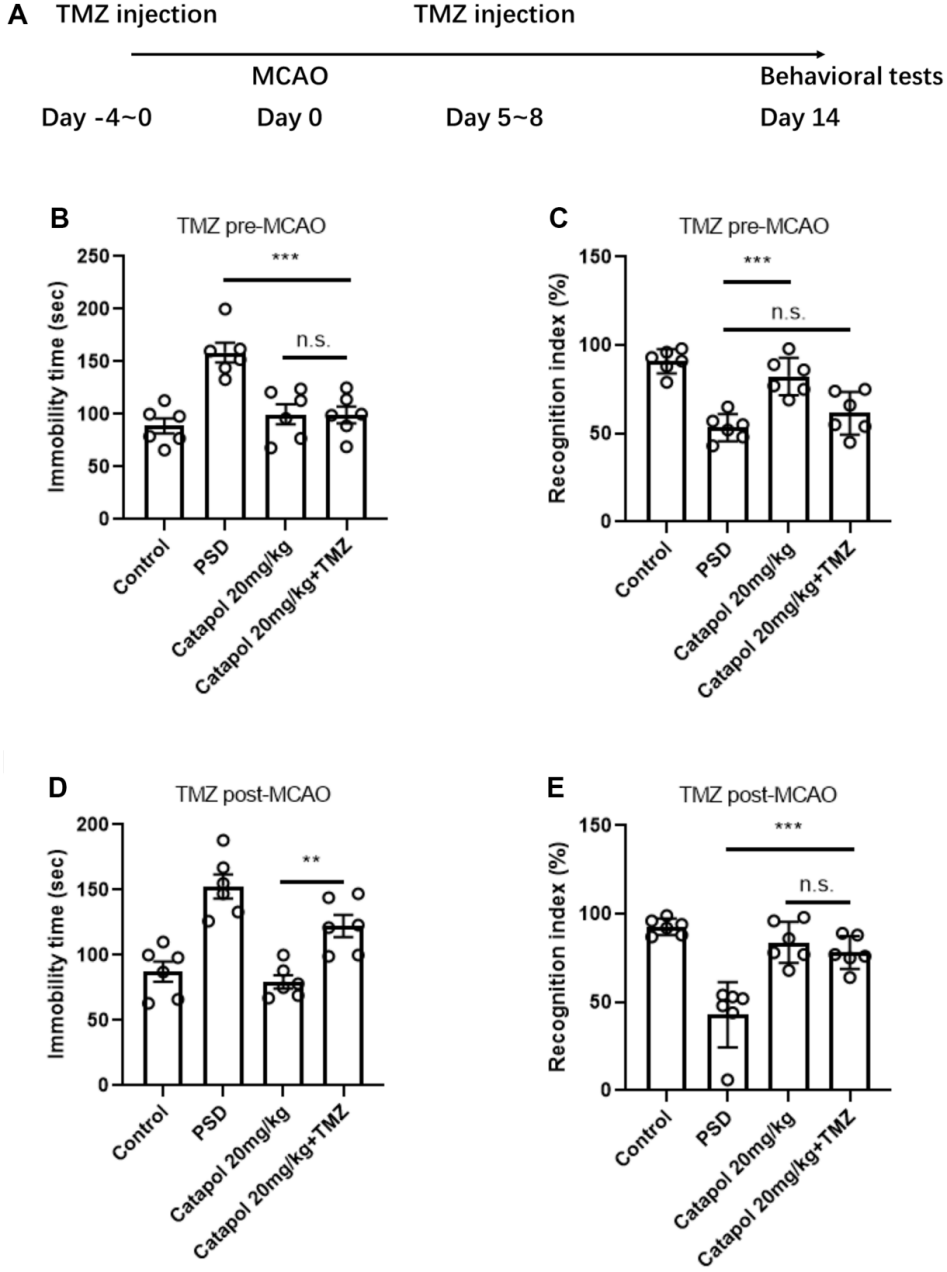
**Catapol perform the behavioral effects in PSD model in dependent with hippocampal neurogenesis.** (**A**) Timeline of the injection procedure of TMZ administration alongside with the drug treatment. (**B**) Statistical analysis on the grooming time in splash test for pre-TMZ treatment. One-way ANOVA, Tukey’s post-hoc, ***: p<0.001. (**C**) Statistical analysis on the recognition index in ORT in pre-TMZ treatment. One-way ANOVA, Tukey’s post-hoc, ***: p<0.001. (**D**) Statistical analysis on the grooming time in splash test for post-TMZ treatment. One-way ANOVA, Tukey’s post-hoc, **: p<0.01. (**E**) Statistical analysis on the recognition index in ORT in post-TMZ treatment. One-way ANOVA, Tukey’s post-hoc, ***: p<0.001.

To confirm whether hippocampal neurogenesis serve as the prerequisite to the behavioral effects of catapol, we treated the catapol administrated PSD mice with temozolomide (TMZ), an imidazotetrazine that capable of blocking DNA replication and lead to cytotoxicity in proliferating cells [16]. TMZ were administrated before and after MCAO performance respectively, which aimed to block the newborn immature neurons and newborn mature neurons [17] (Figure 3A). We applied FST and objective recognition tests to evaluate the antidepressant and cognitive functions. TMZ administration before the MCAO and catapol treatment remarkably affected the recognizing time but did not affect the immobility in FST (Figure 3B, 3C; one-way ANOVA, Tukey’s post-hoc test, B: p<0.001 catapol+TMZ vs. PSD; C: no significance, catapol+TMZ vs. PSD). While administrating the TMZ after MCAO resulted in the insignificant cognitive alteration but remarkable decreased immobility in FST. (Figure 3D, 3E; one-way ANOVA, Tukey’s post-hoc test, B: p<0.01 catapol vs. catapol+TMZ; C: no significance, catapol vs. catapol+TMZ). The result indicates long-term process of neurogenesis could be necessary for the effects of the catapol to cognitive behavior, while short-term immature neurons act as the key mechanism underlying the effects of the catapol to antidepressant behaviors. Therefore, persist neurogenesis in hippocampus perform as the key biological underpinning for the effects of catapol to improve the brain functions in PSD model.

### PI3K signaling plays the key roles in catapol mediated neurogenesis and behavioral effects in PSD

**Figure 4:**
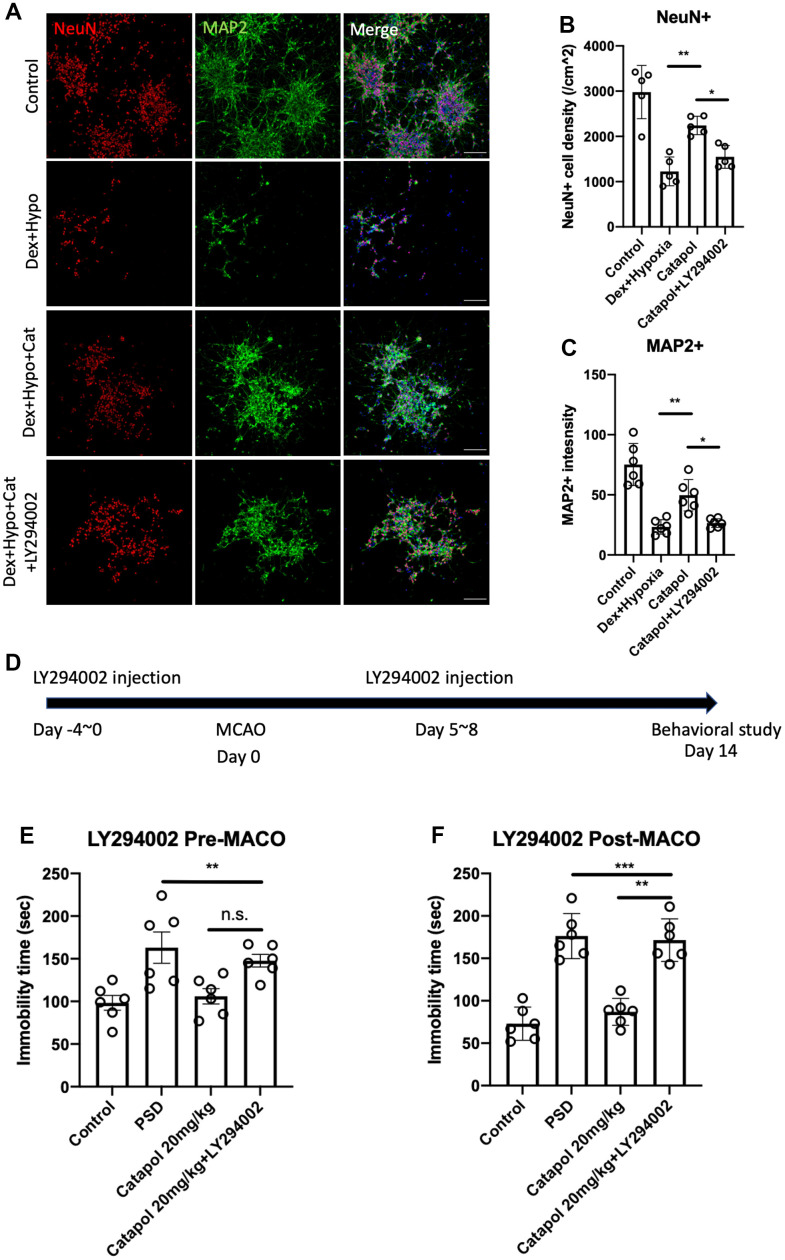
**PI3K signaling plays the key roles in catapol mediated neurogenesis and behavioral effects in PSD.** (**A**) Confocal image to show the primary neuron induction profile under hypoxia and DEX treatment. NeuN was for neural nuclear labelling and MAP2 was used for fiber labelling. (**B**, **C**) Statistical analysis of the NeuN+ and MAP2^+^ neural density under different treatment. One-way ANOVA, post-hoc, *: p<0.05, **: p<0.01. (**D**) Timeline to show the treatment of LY294002 before and after the MCAO model. (**E**, **F**) Statistical analysis to show the immobility time in FST during the LY294002 pre- or post-MCAO administration. One-way ANOVA, post-hoc, **: p<0.01, ***: p<0.001.

Previous study reported that PI3K signaling plays critical roles in the effects of catapol in preventing apoptosis in hydrogen peroxide-induced endothelium [18]. In this study, we used the human derived neural progenitor cells to induce neurons and treated the catapol under hypoxia in combined with agonist of the glucocorticoid receptor dexamethasone (Dex). We used LY294002 the specific PI3K inhibitor to in the group of catapol treatment (Figure 4A). As the results showed, Dex plus hypoxia dramatically decreased the density of the NeuN and MAP2 signals, indicating the dampened neurogenesis *in vitro* (Figure 4B, 4C; one-way ANOVA, Tukey’s post-hoc test, p<0.01 vs. control). Catapol treatment recovered the density of NeuN and MAP2 positive neurons (Figure 4B, 4C; one-way ANOVA, Tukey’s post-hoc test, p<0.01 vs. Dex+Hypo). While administration of LY294002 compromised the effects of catapol to increase new neurons (Figure 4B, 4C; one-way ANOVA, Tukey’s post-hoc test, p<0.01 vs. Dex+Hypo+Cat). This result indicates PI3K signaling plays the key roles in the regulating the pro-neurogenic effects of catapol under PSD condition. To further investigate the biological roles of PI3K signaling in catapol regulated behavioral effects, we next treated LY294002 before and after the MCAO (Figure 3). LY294002 treated before the MCAO model did not result in the significant difference of the mobility time (Figure 4D, 4E; one-way ANOVA, Tukey’s post-hoc test). In contrast, LY294002 treated after the MCAO model significantly increased immobility compared with the catapol administration (Figure 4D, 4F; one-way ANOVA, Tukey’s post-hoc test, p<0.01 catapol vs. catapol+LY294002). Taken together, our results suggest that PI3K signaling plays the key role in regulating neurogenesis and the antidepressant behaviors during the newborn immature neurons formation.

## DISCUSSION

Hippocampal neurogenesis is widely regarded as the key neural plasticity in regulating hippocampus dependent animal behaviors. Brain ischemia induced the neural tissue damage not only results in the acute hippocampal tissue insult but also a long-term stress to depress the hippocampal neurogenesis [19]. In clinical practice, brain functions including emotional behaviors as well as cognitive functions could be improved by treating antidepressants [2]. In current study, we discovered the translational utilization of catapol to improve both cognitive and antidepressant functions in PSD, which was comparable to the effects of commonly used clinical medications piracetam and FLX. Neurogenesis is the prerequisite for the effects of catapol in cognitive and antidepressant improvement. Moreover, PI3K is the key molecular mechanism underlying the effects of catapol in PSD treatment.

The PSD models were commonly performed with the restraint or chronic unpredictable stress following the surgery of MCAO [20, 21]. In current study, we optimized such model to match with the clinical patient’s condition better. Patients after stroke common undergo intrinsic mood stress due to the suffering of the stroke occurrence as well as the panic emotion. Corticosterone long-term feeding has been reported that could induce the depression/anxiety symptoms associating with the declined neurogenesis [17, 22]. Therefore, we used corticosterone treatment after stroke model one for mimic the clinical condition.

Piracetam and FLX are two commonly used clinical drugs for improving brain functions during post-stroke. It was reported that piracetam could improve the generation and the dendritic enrichment of newborn neurons by regulating mitochondrial electronic transport chain (ETC) [23]. On the other hand, by regulating the serotonin transmission, FLX regulate the hippocampal neurogenesis and the relative antidepressant behaviors. In our study, we detected that treatment of catapol enhanced the cognitive behavior and attenuated the depressive emotion as piracetam and FLX treatments (Figure 1). Hippocampal neurogenesis acted as the key regulator to cognitive and antidepressant behaviors. By staining hippocampal tissue with immature neuronal marker DCX, we found that catapol could improve the generation of the immature neurons (Figure 2), which play the key roles in regulating panic memory clearance and enhance the cognitive ability [24]. Since hippocampal neural plasticity includes not only neurogenesis but also synaptogenesis and post synaptic functions, we therefore tested the role of adult neurogenesis in behavioral effects of catapol by treating animal with TMZ. Blockage of the neurogenesis before MCAO resulted in the impaired generation of mature neurons, while treatment of TMZ after MCAO could induce the impaired generation of immature neurons. As noted, enhancement effects of catapol in generation of mature neurons is required for its improvement of cognitive behaviors. The immature neurons formation promoted by catapol is required for the antidepressant function (Figure 3). Consistently, hippocampal mature neurons and immature neurons play distinct roles to animal behaviors. It was reported that immature neurons could establish the GABA-induced excitatory actions that regulate the panic experience separation [25]. Therefore, catapol regulated generation of immature and mature neurons could result in the different types of behavioral effects.

Since the effects of catapol to insulin signaling have been widely reported [12, 26, 27]. Metabolic signaling plays the critical roles in neurogenic regulation [7]. By treating human neural progenitor cells with PI3K inhibitor, we detected the prohibited neural differentiation, indicating PI3K is the key regulator in neurogenesis (Figure 4). By treating PI3K inhibitor before and after the MCAO time point, we observed that only post-treatment of LY294002 could obtained the compromised antidepressant of catapol to PSD model (Figure 4). Therefore, PI3K is the key regulator to the therapeutic effects of catapol to PSD behavioral dysfunctions. However, the roles of other molecular targets that serve as the potential mechanism of catapol cannot be fully explored in current study. More deeper understanding via omics research would be further performed in the future.

To summarize, our research identified the behavioral functions of catapol to PSD behavioral disorders. PI3K mediated adult hippocampal neurogenesis plays the key role in such behavioral effects. This study provided the new insight to harness the natural compounds as the neuroprotective agent to promote brain functional recovery after stroke.
